# No development of ciprofloxacin resistance in the *Haemophilus* species associated with pneumonia over a 10-year study

**DOI:** 10.1186/s12879-015-1267-3

**Published:** 2015-11-13

**Authors:** Josef Yayan, Beniam Ghebremedhin, Kurt Rasche

**Affiliations:** Witten/Herdecke University, Witten, Department of Internal Medicine, Division of Pulmonary, Allergy, and Sleep Medicine, HELIOS Clinic Wuppertal, Heusnerstr. 40, 42283 Wuppertal, Germany; Witten/Herdecke University, Witten, Institute of Medical Laboratory Diagnostics, Center for Clinical and Translational Research, HELIOS Clinic Wuppertal, Wuppertal, Germany

**Keywords:** *Haemophilus influenzae*, *Haemophilus parainfluenzae*, Pneumonia, Antibiotic, Sensitivity, Resistance

## Abstract

**Background:**

The widespread overuse of antibiotics promotes the development of antibiotic resistance in bacteria, which can cause severe illness and constitutes a major public health concern. *Haemophilus* species are a common cause of community- and nosocomial-acquired pneumonia. The antibiotic resistance of these Gram-negative bacteria can be prevented through the reduction of unnecessary antibiotic prescriptions, the correct use of antibiotics, and good hygiene and infection control. This article examines, retrospectively, antibiotic resistance in patients with community- and nosocomial-acquired pneumonia caused by *Haemophilus* species*.*

**Methods:**

The demographic, clinical, and laboratory data of all patients with community- and nosocomial-acquired pneumonia caused by *Haemophilus* species were collected from the hospital charts at the HELIOS Clinic, Witten/Herdecke University, Wuppertal, Germany, within a study period from 2004 to 2014. Antimicrobial susceptibility testing was performed for the different antibiotics that have been consistently used in the treatment of patients with pneumonia caused by *Haemophilus* species.

**Results:**

During the study period of January 1, 2004, to August 12, 2014, 82 patients were identified with community- and nosocomial-acquired pneumonia affected by *Haemophilus* species. These patients had a mean age of 63.8 ± 15.5 (60 [73.2 %, 95 % CI 63.6 %–82.8 %] males and 22 [26.8 %, 95 % CI 17.2 %–36.4 %] females). *Haemophilus* species had a high resistance rate to erythromycin (38.3 %), ampicillin (24.4 %), piperacillin (20.8 %), cefuroxime (8.5 %), ampicillin-sulbactam (7.3 %), piperacillin-sulbactam (4.3 %), piperacillin-tazobactam (2.5 %), cefotaxime (2.5 %), and levofloxacin (1.6 %). In contrast, they were not resistant to ciprofloxacin in patients with pneumonia (*P* = 0.016).

**Conclusion:**

*Haemophilus* species were resistant to many of the typically used antibiotics. Resistance toward ciprofloxacin was not detected in patients with pneumonia caused by *Haemophilus* species.

## Background

*Haemophilus* are rod-shaped, aerobic, facultative anaerobic, Gram-negative coccobacilli belonging to the family *Pasteurellaceae* [1]. All 16 species of *Haemophilus* are motile rods; of these 16 species, the two of medical importance are *H. influenzae* and *H. parainfluenzae. H. influenzae* is sometimes located in the mucous membranes of humans and can cause inflammation in the case of injury to the mucous membranes or immunosuppression [2]. *H. influenzae* is a common cause of bacterial superinfection of the respiratory tract. In particular, it often exacerbates chronic bronchitis in smokers [3].

Amoxicillin-clavulanic acid, azithromycin, clarithromycin, cefaclor, cefprozil, cefixime, and cefuroxime are antimicrobial agents of oral administration that may be used as empiric therapy for respiratory tract infections due to *Haemophilus* species [4]. The results of susceptibility tests with these antimicrobial agents are often not useful for the management of patients with pneumonia caused by *Haemophilus* species. However, susceptibility tests with these compounds may be appropriate for surveillance or epidemiologic studies.

In *H. influenzae*, there is an increasing development of resistance to aminopenicillin and cephalosporins due to the production of beta-lactamases. It may be necessary to resort to the calculated therapy of non-beta-lactams or beta-lactam antibiotics with a beta-lactamase inhibitor [5–7]. Thus, the role of microbiologists is crucial in providing accurate information concerning the susceptibility of clinical isolates in order to help clinicians in the election of antimicrobials and contributes to reducing the rate of antimicrobial resistance emergence.

For this reason, an investigation was conducted to identify the *Haemophilus* species that were resistant to commonly used antibiotics over the last 10 years. Using the hospital database at the HELIOS Clinic, Witten/Herdecke University, in Wuppertal, Germany, data were collected on all the patients with pneumonia. The choice of the correct, effective antibiotic against this Gram-negative bacterium should shorten the duration of patients’ suffering and the length of their hospital stay, as well as reduce mortality.

## Methods

### Patients

This quality-control observational study retrospectively examined the resistance to antibiotics in isolates from patients with diagnosed community- or nosocomial-acquired pneumonia triggered by *Haemophilus* species according to the latest edition of International Classification of Diseases (ICD) code J14 [8, 9]. Data were collected from hospital charts at the HELIOS Clinic, Witten/Herdecke University, in Wuppertal, Germany, during the study period from January 1, 2004, to August 12, 2014. The study population was mixed in terms of age. All patients over 18 years old who were detected to have community- or nosocomial-acquired pneumonia caused by *Haemophilus* species were included in the study. All the patients with nosocomial-acquired pneumonia caused by *Haemophilus* species but who were treated initially for other medical reasons in other departments, such as Internal Medicine and Surgery, were included in this study*.* All the patients examined at the Department of Neurology who had been suspected of having pneumonia caused by *Haemophilus* species were excluded from this study because of restricted access to their patient data.

### Data collection

All patients admitted to this hospital during the above-noted study period who had a microbiological examination of tracheal or bronchial aspirates, blood cultures, or secretion drainage performed for suspected pneumonia were included in this study. We usually noted the patient’s age and sex; the number of tracheal or bronchial aspirates, blood cultures, or secretion drainages collected; the outcome of the culture; the susceptibility and resistance of the isolates to commonly used antimicrobial agents; comorbidities from the hospital records; and inflammatory markers from the blood laboratory values. Data from these records were recorded in spreadsheets using Excel (Microsoft).

### Definition of pneumonia

Pneumonia is an acute inflammation of the lung caused by *Haemophilus* species. Typical clinical symptoms of pneumonia include coughing, chest pain, fever, and difficulty breathing. The diagnosis of pneumonia is performed by X-ray examination and sputum culture [8, 10].

Community-acquired pneumonia is acquired from normal social contact in the community; this is in contrast to nosocomial-acquired pneumonia, which is acquired during hospitalization [11].

The specific criteria that were used for the diagnosis of pneumonia by *Haemophilus* species were that all patients were hospitalized and showed new areas of infiltration upon X-ray examination and novel clinical symptoms, together with a minimum of two of the following: difficulty breathing, temperature over 38 °C, sputum production, and coughing.

### Tested antibiotics

Susceptibility to the following antibiotics was tested against *Haemophilus* species: ampicillin, piperacillin, ampicillin and sulbactam, piperacillin and sulbactam, piperacillin and tazobactam, cefuroxime, cefotaxime, ciprofloxacin, levofloxacin, and erythromycin.

The frequency of use of these antibiotics in clinical practice for the treatment of the study group was recorded. Additionally, the frequency of the testing of these antibiotics on an antibiogram after detecting microbial *Haemophilus* species was noted.

After evaluating the antibiograms*,* the antibiotic that was most commonly used for treatment and most tested for antibiotic susceptibility was compared with the other antimicrobial agents. The antibiotic with the lowest resistance rate was also compared with the other antibiotics tested in the antibiograms. The rates of antibiotic susceptibility and resistance were compared between all isolates from the patients of the study group.

For *Haemophilus* species, inhibition zone diameter breakpoints were used, as recommended in the Clinical and Laboratory Standards Institute (CLSI) 2004–2011 antibiotic susceptibility testing guidelines [12]. In 2011, the Europe-wide standards for susceptibility testing (EUCAST) were adopted instead of CLSI since they take into account the clinical and pharmacokinetic aspects of antimicrobial therapy [13].

### Identification and antimicrobial susceptibility testing

*H. influenzae* was identified based on growth on chocolate agar with bacitracin (Becton Dickinson, Heidelberg) after 18–48 h at 37 °C with 5 % CO_2_ and as oxidase-positive, porphyrin-negative bacteria requiring nicotinamide adenine dinucleotide and heme and lacking beta-hemolysis on horse blood agar, as well as by the use of MALDI-TOF-MS (Bruker, Bremen, Germany). Software suitable for the interpretation of the susceptibility testing results using the EUCAST breakpoints 2012–2014 was used for the antimicrobial susceptibility testing [13]. *H. influenzae* isolates were further confirmed by the use of the API NH system (biochemical reactions) for *Neisseria* and *Haemophilus* identification (bioMérieux, Marcy l'Etoile, France).

Antimicrobial susceptibility testing was performed using the Kirby-Bauer disk diffusion method [14]. In cases of discrepancies or insufficient readings, determination of the minimum inhibitory concentration (MIC) was performed using the E-test for particular antimicrobials, and the results were interpreted according to the EUCAST criteria [13]. Intermediate isolates were grouped with resistant isolates. The antibiotics that were tested against *H. influenzae* isolates are shown in Table 1 [12, 15].

**Table 1:** Zone diameter interpretive standards and equivalent MIC breakpoints for *H. influenzae* and *H. parainfluenzae*

	CLSI 2004–2011					EUCAST 2012–2014			
	MIC breakpoints (μg/mL)		Disk content (μg)	Zone diameter breakpoint (mm)		MIC breakpoints (mg/dL)		Disk content (μg)	Zone diameter breakpoint (mm)
Antibiotic	Sensitive ≤	Resistant ≥				Sensitive ≤	Resistant >		
				Sensitive ≥	Resistant ≤				Sensitive ≥
Ampicillin	1	4	10	22	18	1	1	2	18
Piperacillin	1	4	10	22	18	1	1	2	18
Ampicillin + Sulbactam	2	2	10 + 10	20	19	1	1	10 + 10	Not available
Piperacillin + Sulbactam	1	2	10 + 10	20	19	2	2	10 + 10	Not available
Piperacillin + Tazobactam	1	2	100 + 10	21	–	2	2	Susceptibility inferred from ampicillin susceptibility	
Cefuroxime	4	16	30	20	16	1	2	5	17
Cefotaxime	2	–	30	26	–	0.125	0.125	5	25
Ciprofloxacin	1	–	5	21	–	0.5	0.5	1	28
Levofloxacin	2	–	5	17	–	1	1	1	26
Erythromycin	–	–	–	–	–	0.5	16	5	50

Beta-lactamase production was examined using the nitrocefin test (Oxoid, Wesel, Germany). *H. influenzae* strains were defined as beta-lactamase-negative strains that were resistant to ampicillin (zone diameter >16 mm or MIC ≥4 μg/mL, EUCAST Table) [13]. Inhibition zone diameters were interpreted according to the 2014 EUCAST guidelines [13].

### Disk diffusion (EUCAST standardized disk diffusion method)

The secondary method used for susceptibility testing was the disk diffusion method according to Kirby-Bauer [14]. Antimicrobial susceptibility testing of the *H. influenzae* isolates was performed by the disk diffusion method following the EUCAST guidelines [13]; Mueller-Hinton agar was supplemented with 5 % horse blood and 20 mg/L beta-nicotinamide adenine dinucleotide (BD, Heidelberg, Germany). Plates were inoculated with samples of each isolate and adjusted to a turbidity of 0.5 McFarland. Antibiotic discs were applied to the dried surface of the inoculated agar and further incubated at 35 ± 1 °C for 18 ± 2 h in a 5 % CO_2_ atmosphere.

### Microbiology

The bronchoalveolar lavage was applied in the context of a bronchoscopy. The guidelines and recommendations by the European Respiratory Society Task Force were taken into account for the technical aspects of the bronchoalveolar lavage. Most of the fiber-optic video bronchoscopies used were the OLYMPUS type BF1T180 (Olympus Ltd, Hamburg, Germany) or the high-resolution video bronchoscopy PENTAX type EPK-100p (Pentax Europe Ltd, Hamburg, Germany). In each case, about 20 ml of 0.9 % saline solution were instilled under local anesthesia and aspirated through the fiber-optic bronchoscope again. The aspirate thus obtained was deposited in three different sterile, 40 ml specimen traps (Argyle™ Specimen Traps, Covidien Germany Ltd, Neustadt/Donau, Germany). The quality of the bronchoalveolar lavage was evaluated in order to confirm that it was representative of the alveoli; the criteria used were as follows: volume greater than 20 ml, total cell count greater than 60,000 cells/ml, less than 1 % squamous cells, less than 5 % bronchial epithelial cells, small amounts of debris, and some heavily damaged cell morphology.

Tracheal secretions were also collected by fiber-optic bronchoscopy through aspiration into sterile, 40 ml specimen traps (Argyle™ Specimen Traps, Covidien Germany Ltd, Neustadt/Donau, Germany).

The throat swab was collected using a commercial cotton swab transport system (MEUS Srl®, Piove di Sacco, Italy) by rotating the swab with slight pressure on the palatal arch of the patients. The recovery of sputum was performed by expectoration into a 30 ml sterile sputum collection tube (Salivette®, SARSTEDT, Nümbrecht, Germany).

Sputum and tracheal and bronchial secretions were used for microscopic examination, which was conducted after Gram staining in 80–1000 fold magnification of at least five visual fields according to the criteria of Bartlett [16].

After that, three solid culture media were applied for the cultivation of the most common aerobic, fast-growing microorganisms as a base culture. Columbia Agar with 5 % sheep blood and MacConkey Agar (Becton Dickinson, Heidelberg, Germany) was incubated at 37 °C for 24 to 48 h as a general culture medium for the growth and discovery of *Streptococcus pneumoniae*, *Streptococcus pyogenes*, *Staphylococcus aureus*, *Escherichia coli*, and *Shigella flexneri*.

BD™ Chocolate Agar (Becton Dickinson, Heidelberg, Germany) was used as a variant of blood agar for the isolation and cultivation of *Neisseria* and *Haemophilus* species, in which lysis of the erythrocytes was achieved through a brief heating of the agar at 80 °C.

The BBL™ CHROMagar™ Orientation medium (Becton Dickinson, Heidelberg, Germany) was used for the detection of *Enterobacteriaceae.*

The medium BBL™ CDC Anaerobe 5 % Sheep Blood Agar (Becton Dickinson, Heidelberg, Germany) was used for antimicrobial susceptibility testing for the general growth of anaerobes.

BD™ MacConkey Agar (Becton Dickinson, Heidelberg, Germany) was used as a selective medium for the detection of Gram-negative bacteria.

BD™ Sabouraud Agar (Becton Dickinson, Heidelberg, Germany) and microscopic analysis were used for the identification of fungi.

### Blood cultures

Several blood cultures were employed to detect pathogens that propagate through the blood stream. A minimum of 20 ml of blood was taken through venipuncture with a blood-collection needle (Safety-Multifly®, SARSTEDT, Nümbrecht, Germany) and injected into two specific media—BACTEC Plus Aerobic/F and Plus Anaerobic/F medium (BD, Becton, Dickinson and Company, Heidelberg, Germany).

### Laboratory

After the sample collection, the quantitative determination of C-reactive protein (CRP) in the human serum and plasma (the normal value is less than 6 mg/L) was measured in lithium heparin SARSTEDT Monovette® 4.7 ml (orange top) using a standard immunoturbidimetric assay on the COBAS® 6000 INTEGRA system c 501 (Roche Diagnostics Ltd, Mannheim, Germany). The determination of the leukocyte count (normal range 4000–10,000/μL) in the blood was generally carried out as a routine part of blood counts after collection in EDTA Monovette® 2.7 mL by flow cytometry using the Sysmex® XE 2100 hematology analyzer (Sysmex Germany Ltd, Norderstedt, Germany). CRP and leukocyte counts were compared between all patients in the study group.

### Comorbidities

Comorbidities were analyzed in the study group. Comorbidity was considered to be the presence of one or more additional disorders, which may be a behavioral or mental disorder, existing simultaneously with the primary disease.

Additionally, the lengths of the hospital stays and the number of deaths during hospitalization were determined in the study group. The survival analyses were completed using the Kaplan Meier method; the number of days before discharge from the hospital that death occurred was calculated, and the total number of patients in the study group was considered.

### Ethics statement

The methods of this study were carried out in accordance with the approved institutional guidelines of Witten/Herdecke University in Germany. All the patients’ data were anonymized prior to analysis. The Ethics Committee of Witten/Herdecke University in Germany approved this study and all experimental protocols. Due to the retrospective nature of the study protocol, the Ethics Committee of Witten/Herdecke University in Germany waived the need for written, informed consent.

### Statistical analysis

The categorical data were expressed in proportion, while continuous data were expressed as a mean and standard deviation. The calculations were performed at a 95 % confidence interval (CI) for the sex difference of the patients in the study group. A chi-square test for two independent standard normal variables of three probabilities was carried out to identify whether *Haemophilus* was sensitive, intermediate, or resistant to antibiotics; to compare the effect of antibiotics on patients with community- and nosocomial-acquired pneumonia; and to compare the results using CLSI and EUCAST criteria*.* A chi-square analysis was performed using the VassarStats website for statistical computation, created by Richard Lowry of Vassar College in Poughkeepsie, New York, USA [17]. For the calculation of the *P* value using a 2 × 3 chi-square test, a contingency table was created containing up to two rows and three columns. The rows represented the amount of active substance of the antibiotics that was tested against *Haemophilus*; the antibiotic combination of ampicillin and sulbactam had the highest resistance rate, while ciprofloxacin had no resistance profile when compared with the other antibiotic substances. The three columns were populated by numbers that categorized the *Haemophilus* as sensitive, intermediate, or resistant to the tested antibiotics. One-way analysis of variance (ANOVA) for independent samples was performed to compare, for each antibiotic, the number of samples that were classified as sensitive, intermediate, or resistant to the antibiograms. Two-tailed tests were performed, and a *P* value of less than 0.05 was considered statistically significant.

## Results

In the hospital database used in this study, 140 (2.0 %, 95 % CI 1.7 %–2.3 %) patients were found with pneumonia caused by *Haemophilus* species (ICD J14). This is compared to 6932 patients in all age groups with pneumonia caused by different types of bacteria who had been treated at the HELIOS Clinic, Witten/Herdecke University, Wuppertal, Germany, during the study period from January 1, 2004, to August 12, 2014.

Eighty-two (1.2 %, 95 % CI 0.9 %–1.5 %) of 6932 patients, with a mean age of 63.8 ± 15.5 (60 [73.2 %, 95 % CI 63.6 %–82.8 %] males and 22 [26.8 %, 95 % CI 17.2 %–36.4 %] females), with pneumonia caused by *Haemophilus* species met the inclusion criteria for this trial. The male sex was more likely to suffer from pneumonia caused by *Haemophilus* species.

The patients were divided into categorical groups depending on the origin of their pneumonia caused by *Haemophilus* species. These groups were community-acquired pneumonia, to which 53 patients belonged (64.6 %, 95 % CI 54.3 %–75.0 %); nosocomial-acquired pneumonia, to which 26 patients belonged (31.7 %, 95 % CI 21.6 %–41.8 %); and aspiration pneumonia, to which three patients belonged (3.7 %, 95 % CI 0 %–7.8 %).

Fifty-eight patients were excluded from this study. The reasons for the exclusion of these patients were that they had another infectious disease caused by *Haemophilus* species or that access to their patient data at the Department of Neurology was restricted. In addition, patients with pneumonia caused by *Haemophilus* species that were under the age of 18 and were treated at the Department of Pediatric and Adolescent Medicine were excluded.

The number of tests for each antibiotic varied in this study because some isolates were examined according to the CLSI guidelines, while in more recent years, others were examined according to the EUCAST guidelines. In general, the number of antimicrobial susceptibility tests using the CLSI guidelines was higher (Table 2). A comparison of susceptibility testing between the CLSI and EUCAST criteria showed more resistance rates in *Haemophilus* species to erythromycin using the EUCAST criteria (*P =* 0.002; Table 2).

**Table 2:** Antimicrobial susceptibility of *Haemophilus* species from patients with pneumonia. Comparison of antimicrobial susceptibility between CLSI 2004–2011 and EUCAST 2012–2014 criteria

			Number of patients with *Haemophilus* species = 82						
Drug groups	Active substance	No. using antibiotics (%)	No. of antibiotic tests on antibiogram (%)	Sensitive (%)	Inter-mediate (%)	Resistant (%)	*P* value compared with Ampicillin + Sulbactam	*P* value compared with Ciprofloxacin	*P* value CAP compared to NAP
Penicillins	Ampicillin	1 (1.2)	82 (100)	61 (74.4)	1 (1.2)	20 (24.4)	**0.011**	**<0.0001**	
	CAP	0	53 (100)	38 (71.7)	1 (1.9)	14 (26.4)	**0.010**	**<0.0001**	0.730
	NAP	1 (3.8)	26 (100)	20 (76.9)	0	6 (23.1)	**0.066**	**<0.0001**	0.730
	CLSI	1 (2.9)	34 (100)	24 (70.6)	1 (2.9)	9 (26.5)	**0.019**	**<0.0001**	
	EUCAST	0	48 (100)	38 (79.2)	0	11 (22.9)	**0.028**	**<0.0001**	
	Piperacillin	1 (1.2)	24 (29.3)	19 (79.2)	0	5 (20.8)	0.128	**0.0001**	
	CAP	1 (1.9)	12 (22.6)	8 (66.7)	0	4 (33.3)	**0.022**	**<0.0001**	0.430
	NAP	0	10 (38.5)	9 (90.0)	0	1 (10.0)	0.848	**0.0167**	0.430
	CLSI	1 (2.9)	24 (70.6)	19 (79.2)	0	5 (20.8)	0.128	**0.0001**	
	EUCAST	0	0	0	0	0	1.0	1.0	
Penicillin + Beta- lactamase inhibitors	Ampicillin + Sulbactam	28 (34.1)	82 (100)	74 (90.2)	2 (2.4)	6 (7.3)		**0.016**	
	CAP	23 (43.4)	53 (100)	47 (88.7)	2 (3.8)	4 (7.5)	0.905	**0.008**	0.604
	NAP	4 (15.4)	26 (100)	24 (92.3)	0	2 (7.7)	0.723	**0.042**	0.604
	CLSI	12 (35.3)	34 (100)	29 (85.3)	2 (5.9)	3 (8.8)	0.619	**0.002**	
	EUCAST	16 (33.3)	48 (100)	45 (93.8)	0	3 (6.3)	0.533	0.075	
	Piperacillin + Sulbactam	8 (9.8)	23 (28.0)	22 (95.7)	0	1 (4.3)	0.651	0.169	
	CAP	2 (3.8)	11 (20.8)	10 (90.9)	0	1 (9.1)	0.856	**0.024**	0.622
	NAP	4 (15.4)	10 (38.5)	10 (100)	0	0	0.586	1.0	0.622
	CLSI	8 (23.5)	23 (67.6)	22 (95.7)	0	1 (4.3)	0.651	0.169	
	EUCAST	0	0	0	0	0	1.0	1.0	
	Piperacillin + Tazobactam	24 (29.3)	79 (96.3)	77 (97.5)	0	2 (2.5)	0.135	0.354	
	CAP	16 (30.2)	51 (96.2)	49 (96.1)	0	2 (3.9)	0.375	0.199	0.592
	NAP	8 (30.8)	26 (100)	26 (100)	0	0	0.254	1.0	0.592
	CLSI	8 (23.5)	31 (91.2)	29 (93.5)	0	2 (6.5)	0.667	0.070	
	EUCAST	16 (33.3)	48 (100)	48 (100)	0	0	0.083	1.0	
Cephalosporins	Cefuroxime	3 (3.7)	82 (100)	73 (89.0)	2 (2.4)	7 (8.5)	0.961	**0.009**	
	CAP	0	53 (100)	46 (86.8)	1 (1.9)	6 (11.3)	0.715	**0.004**	0.492
	NAP	3 (11.5)	26 (100)	24 (92.3)	1 (3.8)	1 (3.8)	0.771	**0.042**	0.492
	CLSI	3 (8.8)	34 (100)	28 (82.4)	1 (2.9)	5 (14.7)	0.454	**0.0005**	
	EUCAST	0	48 (100)	45 (93.8)	1 (2.1)	2 (4.2)	0.760	0.075	
	Cefotaxime	0	80 (97.6)	78 (97.5)	0	2 (2.5)	0.13	0.359	
	CAP	0	52 (98.1)	50 (96.2)	0	2 (3.8)	0.362	0.206	0.598
	NAP	0	26 (100)	26 (100)	0	0	0.254	1.0	0.598
	CLSI	0	32 (94.1)	31 (96.9)	0	1 (3.1)	0.461	0.279	
	EUCAST	0	48 (100)	47 (97.9)	0	1 (2.1)	0.235	0.427	
Gyrase inhibitors	Ciprofloxacin	5 (6.1)	81 (98.8)	81 (100)	0	0	**0.016**		
	CAP	2 (3.8)	52 (98.1)	52 (100)	0	0	0.067	1.0	1.0
	NAP	2 (7.7)	26 (100)	26 (100)	0	0	0.254	1.0	1.0
	CLSI	2 (5.9)	33 (97.1)	33 (100)	0	0	0.177	1.0	
	EUCAST	3 (6.3)	48 (100)	48 (100)	0	0	0.083	1.0	
	Levofloxacin	4 (4.9)	63 (76.8)	62 (98.4)	0	1 (1.6)	0.121	0.525	
	CAP	4 (7.5)	46 (86.8)	46 (100)	0	0	0.091	1.0	0.253
	NAP	0	17 (65.4)	16 (94.1)	0	1 (5.9)	0.787	0.090	0.253
	CLSI	0	16 (47.1)	16 (100)	0	0	0.427	1.0	
	EUCAST	4 (8.3)	47 (97.9)	46 (97.9)	0	1 (2.1)	0.244	0.419	
Macrolide	Erythromycin	0	81 (98.8)	43 (53.1)	7 (8.6)	31 (38.3)	**<0.0001**	**<0.0001**	
	CAP	1 (1.9)	52 (98.1)	27 (51.9)	6 (11.5)	19 (36.5)	**<0.0001**	**<0.0001**	0.458
	NAP	0	26 (100)	13 (50)	1 (3.8)	12 (46.2)	**<0.0001**	**<0.0001**	0.458
	CLSI	1 (2.9)	33 (97.1)	24 (72.7)	4 (12.1)	5 (15.2)	**0.037**	**<0.0001**	
	EUCAST	0	48 (100)	19 (39.6)	3 (6.3)	26 (54.2)	**<0.0001**	**<0.0001**	

Results of susceptibility testing of the active substances are shown in bold according to both CLSI and EUCAST criteria. Results are also shown to compare CAP and NAP

*Abbreviations*: *CAP* community-acquired pneumonia, *CLSI* Clinical and Laboratory Standards Institute, *EUCAST* Europe-wide standards for susceptibility testing, *NAP* nosocomial-acquired pneumonia, *MIC* minimum inhibitory concentration

Significant *P* values shown in bold

There were highly significant differences with regard to the number of samples classified as either sensitive, intermediate, or resistant to a particular antibiotic within this study group (*P* <0.0001). In the susceptibility testing, the mean numbers of samples tested against antibiotics that were classified as sensitive, intermediate, and resistant were 59 ± 23.2, 1.2 ± 2.2 and 7.5 ± 10.1, respectively (Table 2).

The most-administered antibiotics in these patients were the combination of ampicillin and sulbactam, followed by piperacillin-tazobactam and piperacillin-sulbactam combinations (Table 2).

No resistance was found to ciprofloxacin in any of the patients in this study group compared to ampicillin-sulbactam; this finding is statistically significant (*P* = 0.016; Table 2).

*Haemophilus* species had the highest resistance rate to erythromycin compared to both ampicillin-sulbactam and ciprofloxacin in this study (*P <*0.0001; Table 2). *Haemophilus* species also had a high resistance rate to ampicillin compared with ampicillin-sulbactam in this investigation (*P* = 0.011; Table 2). The statistical comparison of ampicillin, with the highest rate of resistance, to ciprofloxacin, with no resistance rate, was also determined in this study (*P* <0.0001; Table 2). When the amount of each antibiotic actually tested was taken into consideration, *Haemophilus* species showed high resistance rates to erythromycin, ampicillin, piperacillin, cefuroxime, ampicillin-sulbactam, piperacillin-sulbactam, piperacillin-tazobactam, cefotaxime, and levofloxacin (Table 2). Again, when considering the amount of each antibiotic that was actually tested, *Haemophilus* species were most sensitive to the following antibiotics, in order of decreasing effectiveness: ciprofloxacin, levofloxacin, cefotaxime, piperacillin-tazobactam, piperacillin-sulbactam, ampicillin-sulbactam, cefuroxime, piperacillin, ampicillin, and erythromycin (Table 2).

The susceptibility and resistance rates of antibiotics were not significantly different when comparing patients with community-acquired pneumonia and patients with nosocomial-acquired pneumonia (Table 2).

*Haemophilus* species were most detected in tracheal secretions (Table 3). More than half of the discovered *Haemophilus* species were from isolates of *H. influenzae* (Table 3)*.* One patient had both species in their bronchial secretions.

**Table 3:** Clinical specimens and species of *Haemophilus* from patients with pneumonia

Specimen	No. of patients (%)	95 % CI %
Bronchial secretion	25 (30.5)	20.5–40.5
Tracheal secretion	30 (36.6)	26.2–47.0
Sputum	22 (26.8)	17.2–36.4
Throat swab	1 (1.2)	0–3.6
Arterial blood culture	0	0
Venous blood culture	9 (11.0)	4.2–17.8
Secretion drainage	1 (1.2)	0–3.6
Species		
*Haemophilus influenzae*	46 (56.1)	45.4–66.8
*Haemophilus parainfluenzae*	37 (45.1)	34.3–55.9

Several tests were carried out partially in some patients. Both *H. influenzae and H. parainfluenzae* were found in one patient’s bronchial secretions

The amount of CRP in the serum and plasma of the study group patients had a mean value of 98.3 mg/L ± 109.5 mg/L. The leukocyte count in the blood of the study group patients had a mean value of 12,729.9/μL ± 6345.9/μL. There was no difference in the level of CRP between patients with community-acquired pneumonia (103.8 mg/L ± 121.9 mg/L) and nosocomial-acquired pneumonia (78.9 mg/L ± 84.4 mg/L) (*P* = 0.351). Likewise, the leukocyte counts did not differ between patients with community-acquired pneumonia (12,455.2/μL ± 7168.3/μL) and nosocomial-acquired pneumonia (12,289.2/μL ± 5388.7/μL) (*P =* 0.921).

In this study group, most of the identified comorbidities were cardiac arrhythmias, acute respiratory failure sepsis, and myocardial infarction (Table 4). The common chronic comorbidities were hypertension, chronic obstructive pulmonary disease, coronary artery disease, and diabetes (Table 4). The length of the hospital stay of these patients had a mean of 12.1 ± 8.2 days. There were 10 (12.2 %, 95 % CI 5.1 %–19.3 %) deaths associated with pneumonia caused by *Haemophilus* species*.* Thus, the survival rate was 87.8 % (95 % CI 80.2 %–95.4 %) in this study group.

**Table 4:** Acute and chronic comorbidities in patients with pneumonia caused by *Haemophilus* species

Organs	Acute comorbidities	Organs	Chronic comorbidities
Cardiovascular diseases	Number of patients (%)	Cardiovascular diseases	Number of patients (%)
Anemia	8 (9.8)	Aneurysm	3 (3.7)
Angina pectoris	1 (1.2)	Cardiomyopathy	3 (3.7)
Blood pressure derailment	1 (1.2)	Coronary artery disease	20 (24.4)
Cardiac decompensation	9 (11.0)	Heart failure	12 (14.6)
Cardiac arrhythmia	20 (24.4)	Hypertension	41 (50.0)
Cardiopulmonary resuscitation	8 (9.8)	Hypertensive heart disease	4 (4.9)
Deep vein thrombosis	1 (1.2)	Peripheral arterial occlusive disease	5 (6.1)
Myocardial infarction	9 (11.0)	State after heart attack	5 (6.1)
Sepsis	11 (13.4)	Valvular heart disease	4 (4.9)
Shock	3 (3.7)		
Syncope	1 (1.2)		
Pulmonary diseases		Pulmonary diseases	
Acute respiratory distress syndrome	1 (1.2)	Asbestosis	2 (2.4)
Acute respiratory failure	16 (19.5)	Asthma	3 (3.7)
Exacerbation by chronic obstructive pulmonary disease	8 (9.8)	Chronic obstructive pulmonary disease	27 (32.9)
Pleural effusion	8 (9.8)	Chronic respiratory insufficiency	1 (1.2)
Pneumothorax	1 (1.2)	Cor pulmonale	3 (3.7)
Pulmonary embolism	4 (4.9)	Emphysema	12 (14.6)
		Lung fibrosis	1 (1.2)
		Lung tumor	8 (9.8)
		Obstructive sleep apnea syndrome	10 (12.2)
		State after tuberculosis	1 (1.2)
Gastrointestinal diseases		Gastrointestinal diseases	
Cholecystitis	1 (1.2)	Appendectomy	1 (1.2)
Gastritis	2 (2.4)	Cholecystectomy	2 (2.4)
Gastroenteritis	4 (4.9)	Chronic pancreatitis	2 (2.4)
Gastrointestinal bleeding	2 (2.4)	Colitis	1 (1.2)
Hyperglycemia	1 (1.2)	Crohn’s disease	1 (1.2)
Hypoglycemia	1 (1.2)	Diabetes	18 (22.0)
		Diverticulosis	4 (4.9)
		Gallstones	3 (3.7)
		Hemorrhoids	1 (1.2)
		Hiatal hernia	1 (1.2)
		Hyperlipidemia	12 (14.6)
		Liver cirrhosis	1 (1.2)
		Obesity	8 (9.8)
Kidney diseases		Kidney diseases	
Acute renal failure	5 (6.1)	Benign prostatic hyperplasia	2 (2.4)
Acute urinary tract infection	5 (6.1)	Chronic renal failure	6 (7.3)
Electrolyte imbalance	7 (8.5)	Diabetic nephropathy	2 (2.4)
Exsiccosis	1 (1.2)	Prostate carcinoma	1 (1.2)
Urinary retention	1 (1.2)	Renal cyst	1 (1.2)
Urosepsis	1 (1.2)		
Thyroid disease		Thyroid disease	
Hypothyroidism	5 (6.1)	Goiter	2 (2.4)
Orthopedics		Orthopedics	
Fracture	4 (4.9)	Osteoarthritis	2 (2.4)
Traumatic brain injury	1 (1.2)	Osteoporosis	3 (3.7)
		Rheumatism	3 (3.7)
		Spondylosis	1 (1.2)
Ear, nose, and throat diseases		Ear, nose, and throat disease	
Acute rhinosinusitis	3 (3.7)	Presbycusis	1 (1.2)
Laryngitis	1 (1.2)		
Nosebleed	1 (1.2)		
Neurology diseases		Neurology diseases	
Hypoxic encephalopathy	1 (1.2)	Epilepsy	8 (9.8)
Rhabdomyolysis	1 (1.2)	Polyneuropathy	3 (3.7)
Stroke	3 (3.7)	Restless legs syndrome	6 (7.3)
		State after encephalitis	1 (1.2)
		State after stroke	6 (7.3)
		Sturge-Weber syndrome	1 (1.2)
Psychiatric diseases		Psychiatric diseases	
Delirium	4 (4.9)	Alcoholism	3 (3.7)
Depression	2 (2.4)	Dementia	3 (3.7)
		Former smoking	4 (4.9)
Ophthalmological disorder		Ophthalmological disorder	
Retinal detachment	1 (1.2)	State after central retinal vein occlusion	1 (1.2)
Skin diseases		Skin disease	
Allergy	4 (4.9)	Psoriasis	1 (1.2)
Erysipelas	1 (1.2)		
Exanthem	1 (1.2)		

## Discussion

During the 10-year study period in this qualitative control observational study, *Haemophilus* species did not develop resistance to ciprofloxacin, an antibiotic used for the treatment of patients with pneumonia. Ciprofloxacin is a notably effective bactericidal against Gram-negative organisms. Accordingly, the treatment of acute bacterial pneumonias with high-dose parenteral ciprofloxacin was shown to be an efficacious and well-tolerated treatment in a previous Italian study [18]. In another study, ciprofloxacin administered alone or in combination was found to be effective in treating pneumonia when compared with a standard beta-lactam monotherapy or the combination of an aminoglycoside plus a beta-lactam [19].

Levofloxacin was also found to be effective in the present study for the treatment of patients with pneumonia caused by *Haemophilus* species. The development of resistance to levofloxacin was very low over the 10 years in which this study was conducted. The efficacy and safety of levofloxacin in patients with pneumonia has also been demonstrated in a previous study, which found levofloxacin to be effective in the treatment of patients with pneumonia, all of whom tolerated levofloxacin well [20]. However, other studies have reported an increase in cases of levofloxacin resistance in *H. influenzae* [21, 22]*.* Although fluoroquinolone resistance in *H. influenzae* remains rare, these studies reported that their clinical and molecular investigations of levofloxacin-resistant isolates showed that the increase was mainly the result of the spread of several clones in the elderly population in the different regions of study [21, 22]*.*

*H. influenzae* does demonstrate resistance to cefotaxime [23]. The first plasmid-mediated beta-lactamase in Gram-negative bacteria was discovered in Greece in the 1960s. It was named TEM, after the patient from whom it was isolated, Temoniera [24]. Resistance to beta-lactams in *H. influenzae* is mostly due to the presence of TEM beta-lactamases and beta-lactamase-negative ampicillin resistance [24]. It is also known that the beta-lactamase non-producing ampicillin resistant *H. influenzae* strains had their penicillin-binding protein 3 changed, causing them to become beta-lactam non-susceptible during the patients’ treatment. In these cases, CLSI recommended reporting these strains as resistant [12, 25].

With its beta-lactamase inhibitor, the piperacillin-tazobactam combination improves the activity of penicillin against many Gram-positive and Gram-negative pathogens [26]. High efficacy and a low rate of resistance were observed for piperacillin-tazobactam in the present study. An earlier study showed the clinical and bacteriological efficacy and safety of piperacillin-tazobactam in the treatment of adult patients with lower respiratory tract infections needing hospitalization [27]. The data of this earlier study suggested that piperacillin-tazobactam was a reliable therapy for adult patients with severe infections of the lower respiratory tract [27]. Piperacillin-tazobactam was also the most effective beta-lactam tested against *H. influenzae* isolates in the results of another study [28]. A disk diffusion breakpoint for piperacillin-tazobactam of greater than 21 mm was proposed, which we can confirm from the results of our study.

Piperacillin and sulbactam, another combination of the penicillin and beta-lactamase inhibitor classes, showed acceptable results in the susceptibility testing of the patients in our study group. The most recent Canadian and American guidelines for treatment of the above-mentioned infections recommend the use of a combination therapy with beta-lactams and a new generation macrolide or respiratory fluoroquinolone [29]. In the present study, the efficacy and rate of resistance of the antibiotic ampicillin-sulbactam combination were ranked behind the piperacillin and beta-lactam combination.

A previous randomized prospective study compared ampicillin-sulbactam and cefamandole in the therapy of patients hospitalized with community-acquired pneumonia, and ampicillin-sulbactam was shown to be an effective agent for the treatment of community-acquired pneumonia [30]. Another study compared the clinical usefulness of piperacillin therapy to ampicillin-sulbactam for community-acquired pneumonia [31]. In that study, the clinical efficiency of the piperacillin therapy was comparable to that of the ampicillin-sulbactam therapy. The results proposed that piperacillin therapy had good efficiency and tolerability and that piperacillin was highly effective in cases of pneumonia [31]. However, in our study, ampicillin-sulbactam was superior compared with piperacillin in the susceptibility testing of the patients with pneumonia caused by *Haemophilus* species.

Compared to the first-generation cephalosporin antibiotics, cefuroxime has an increased effect on Gram-negative rods, especially against *H. influenzae.* Cefuroxime has a high stability toward beta-lactamase enzymes and has been recommended for the treatment of pneumonia. A study that compared the efficacy and safety of cefuroxime versus amoxicillin-clavulanic acid in the treatment of community-acquired pneumonia found that compared to amoxicillin-clavulanic acid, cefuroxime had comparable efficacy and safety [32]. Cefuroxime axetil, an oral cephalosporin, was approved as an antibiotic with broad-spectrum in vitro antibacterial activity against the beta-lactamase positive respiratory pathogen *H. influenzae* [33]. Clinical studies have evaluated cefuroxime axetil for the treatment of upper and lower respiratory tract infections, and it has demonstrated similar efficacy to established antibacterial agents, including amoxicillin-clavulanic acid and cefaclor [33]. However, the effect of cefuroxime was low against *Haemophilus* species in patients with pneumonia in this study, which the medical literature has long acknowledged [34].

Piperacillin has the broadest activity spectrum of all the penicillins, including *Pseudomonas*, Enterobacteriaceae, Gram-negative rods, and anaerobes [35]. To demonstrate its efficacy, a study examined the clinical usefulness of piperacillin therapy compared to ampicillin-sulbactam for community-acquired pneumonia. The general clinical efficiency of piperacillin therapy in these patients was similar to that of ampicillin-sulbactam therapy. The previous study by Seki et al. concluded that piperacillin was effective in the treatment of pneumonia and proposed piperacillin as a first-line treatment for community-acquired pneumonia [36]. From the results of our study, piperacillin cannot be recommended as a first-line treatment for patients with pneumonia caused by *Haemophilus* species because of its relatively high resistance rate and lower sensitivity rate according to our susceptibility testing results.

Ampicillin is a broad-spectrum antibiotic used against *H. influenzae* [37]. The ampicillin resistance of *H. influenzae* has long been recognized [38]. *H. influenzae* was assumed generally susceptible to ampicillin in patients with pneumonia. Ampicillin resistance in *H. influenzae* isolated from specimens of pneumonia patients is increasing, with resistance rates of 6.6 to 48 % from one institution to another [39]. Most of the resistant *H. influenzae* isolates produce beta-lactamase enzymes. Therefore, it is important for physicians to know their institution’s incidence rate of ampicillin-resistant *H. influenzae* when choosing an empiric therapy for nosocomial-acquired and community-acquired pneumonia. The resistance rate for ampicillin was 24.4 % in this study’s patient group. Patients with *H. influenzae* pneumonia who fail to respond to ampicillin, or who are known to be infected with ampicillin-resistant isolates based on laboratory findings, should receive therapy designed to combat ampicillin-resistant isolates [40].

The antimicrobial spectrum of macrolides is similar to the antibiotic class of penicillin [41]. Erythromycin is a macrolide that is useful in the treatment of a number of bacterial infections [42]. However, *Haemophilus* species in this study demonstrated the highest resistance rate to erythromycin. Macrolides are increasingly used for the treatment of respiratory infections caused by *H. influenzae*, but their usefulness is impaired by the development of resistance in *H. influenzae*. This development of resistance to antibiotics is a growing problem among respiratory pathogens, and the rate of beta-lactam resistance is increasing in *H. influenzae* [43]. Other research has acknowledged that the increased use of macrolides in patients with pneumonia is a reason for the increasing development of resistance in *H. influenzae* to macrolides [44].

Medical professionals can preserve the efficacy of existing antibiotics against *H. influenzae* isolates by administering these only for bacterial infections according to guidelines and antimicrobial stewardship and in the proper dose for the correct therapy duration.

This study used CRP, which is a beneficial biomarker for the diagnosis of community-acquired pneumonia, for diagnosis. The initial values of CRP were not high, with very few exceptions, in the patients of this study group. CRP values did not differ between the patients with community-acquired and nosocomial-acquired pneumonia. CRP is particularly elevated in community-acquired pneumonia caused by *S. pneumoniae* and *L. pneumophila*, which could be useful in differentiating from a diagnosis of community-acquired pneumonia caused by *H. influenzae*. Furthermore, initial levels of CRP reveal the severity of the community-acquired pneumonia and the need for hospitalization in the general ward or intensive care unit [45].

Another factor in the severity of pneumonia is white cell count, which a previous study found to be predictive [46]. In our study, white cell counts were slightly elevated in the patients with *H. influenzae* pneumonia. In comparison to the previous study, the leukocyte counts were different in the blood of the study group patients in the present study. The leukocyte count in this study had a mean value of 12,729.9/μL ± 6345.9/μL compared to 19,800/μL ± 9500/μL in the previous study [46]. The previous study involved only adult inpatients with community-acquired pneumonia. The researchers examined the relations between the levels of CRP, leukocyte count, and erythrocyte sedimentation rate and the severity of pneumonia according to the criteria of different guidelines. Accordingly, medical professionals should consider white cell counts for the diagnosis of pneumonia; however, the increase of white cells also takes place in many other clinical scenarios.

### Study limitations

This study describes the situation of *Haemophilus* resistance in a single hospital, so we cannot generalize the results to other geographic locations. Another limitation of the study was the low number of study patients, with only 82 cases identified by *Haemophilus* over 10 years. After the evaluation of this study, it became apparent that not all antibiotics were tested with the same frequency in the antibiograms of patients with pneumonia caused by *Haemophilus*. The authors were unable to clarify whether or not all of these antibiotics were tested for each *H. influenza* isolate.

## Conclusions

All *Haemophilus* isolates from patients with pneumonia showed resistance to a variety of antibiotics, but none of the patients in the study group showed resistance to ciprofloxacin. In the case of the identification of *Haemophilus* species on culture media from a patient with pneumonia, all common antibiotics, such as those in this study, should be tested for susceptibility in order to determine the most appropriate treatment and to monitor the trend in antibiotic resistance of *Haemophilus* species in the future.

## Abbreviations

CAP: Community-acquired pneumonia
CI: Confidence interval
CLSI: Clinical and Laboratory Standards Institute
CRP: C-reactive protein
EUCAST: Europe-wide standards for susceptibility testing
ICD: International Classification of Diseases
MIC: Minimum inhibitory concentration
NAP: Nosocomial-acquired pneumonia
